# An examination of the Social Skills Improvement System-Rating Scale (SSIS-RS) teacher and parent forms factor structure in a sample of Mexican American preschool-aged children

**DOI:** 10.1371/journal.pone.0329576

**Published:** 2025-08-20

**Authors:** Hanjoe Kim, Jacqueline R. Anderson, Jorge E. Gonzalez

**Affiliations:** 1 Department of Psychology, Yonsei University, Seoul, South Korea; 2 College of Education and Behavioral Sciences, University of Northern Colorado, Greely, Colorado, United States of America; 3 Department of Education, Trinity University, San Antonio, Texas, United States of America; University of Johannesburg Faculty of Education, SOUTH AFRICA

## Abstract

Social skills assessment has become integral to childhood mental health evaluation and a target for interventions, especially among school psychologists. One popular multi-rater social skill instrument is the Social Skills Improvement System Rating Scales (SSIS-RS), which is designed to evaluate social skills, problem behaviors, and academic competence in children. While previous versions of the SSIS have been widely used in research and practice, including use with translations across multiple languages, few studies have examined the psychometric properties of the most recent iteration of the SSIS-RS with diverse populations. The current study used the Spanish Parent and English Teacher versions to assess its construct validity with a Mexican American population. Confirmatory factor analysis conducted with data from 624 parents and 135 teachers rating 761 Mexican American children’s social skills and problem behaviors revealed adequate psychometric properties but varied somewhat from the proposed scale models of the SSIS-RS. The differences may be due to the cultural uniqueness of Mexican origin compared to the norming sample. Further, similarities and discrepancies were found when comparing the factor structures between parent and teacher ratings. These results suggest that while parents and teachers shared views on the children, environmental factors and the language of the SSIS-RS might have influenced their responses.

## Introduction

Social skills encompass a wide array of skills, including initiating conversations, listening and responding appropriately, empathizing, negotiating, and understanding social cues [1,2]. Social skills in early childhood play a key role in predicting children’s later socioemotional and academic success [3]. Indeed, a child’s ability to appropriately express or inhibit social behaviors can impact their development into adulthood [4]. As a result, deficits in social skills can play a key role in a student’s social adjustment in and outside of school [5]. In addition, social skills can be predictive of a broad range of disorders and adverse long-term outcomes such as school dropout or juvenile delinquency [6–8]. For example, both initial and transitional-period assessments of social skills are excellent predictors of early childhood challenges, including autism, conduct disorder, attention deficit/hyperactivity disorder, and emotional and behavioral disorder [9–13].

In acknowledging the influence of social skills on child development, researchers and practitioners alike have increasingly focused on systematic assessment of these abilities [14]. As a result, assessment of social skills has emerged as an essential component in evaluation and intervention strategies among school psychologists and other assessment personnel aimed at promoting childhood wellbeing. Within this context, behavior rating scales are essential tools, offering valuable insights into both a child’s social competencies and deficits. With approximately 97% of school psychologists in a recent survey [15] reporting using behavior rating scales in their practice, the use of such scales across populations and subpopulations warrants further investigation [15].

School psychologists spend approximately 58% of their time addressing the needs of special education students, mainly at the elementary school level [15]. Along with educational diagnosticians and educators, they frequently utilize broad-based behavior rating scales to measure social and related skills. Indeed, use estimates suggest that behavior rating scales, including those that tap into social and related skills, account for about 9% of school psychologists’ comprehensive evaluation tests [15]. Assessments such as the Achenbach System of Empirically Based Assessment Child Behavior Checklist Ages 1–5 ½ [16], the Behavioral Assessment System for Children [17], and the Social Skills Improvement System Rating Scales [14] are foundational in this context, enabling targeted interventions that promote holistic student development within educational environments [2].

Most social skill measures rely on input from multiple raters, such as parents, teachers and, when appropriate, self-reports [18]. However, while such a broad-based approach is generally helpful, differences in perspectives among informants, influenced by factors like race and ethnicity, can pose serious challenges in synthesizing assessment data [5]. Specifically, such discrepancies can introduce systematic biases, potentially resulting in over- or underestimation of specific behavioral traits, thereby complicating the assessment process and result in negative outcomes for children [19].

### The Social Skills Improvement System Rating Scales

One popular multi-informant instrument for assessing social skills is the Social Skills Improvement System Rating Scales (SSIS-RS) [14], an update to the initial version, the Social Skills Rating System (SSRS) [20], which was designed more than 20 years ago to be a brief, low-cost, comprehensive assessment of a child’s social skills from ages 3–18. Both forms have been translated into several languages, including Korean and Spanish, and have demonstrated robust psychometric properties across diverse populations [21,22]. Specifically, several studies have provided empirical support for the reliability and validity of these translated versions [23–27]. Of relevance to the present study, Jurado and colleagues [24] examined the reliability and validity of the early version of the SSRS Spanish version teacher form, specifically internal consistency, test-retest reliability, parent-teacher cross-informant correlations, and construct validity, with a Latinx population. In the study, Puerto Rican teachers rated Puerto Rican elementary students on social skills. Results supported the psychometric properties of the Spanish version of the SSRS teacher form for a Latinx population.

The SSIS-RS updated the original norm sample to reflect a more recent, 2006, U.S. Census sample. The update included linkage from the scales and subscales to interventions such as social skills interventions for children with deficits in social skills [14,22]. Like its predecessor, the SSIS-RS can be administered to children ages 3–18 years [14] and continues to consist of three main domains: social skills, problem behaviors, and academic competence. Social skills constitute a set of learned behaviors that facilitate effective and appropriate interactions within various social contexts. These competencies are typically acquired through processes such as modeling, practice, and reinforcement. Problem behaviors can interfere with the acquisition and execution of socially competent behaviors. Problem behaviors (e.g., aggression, impulsiveness, social withdrawal) can undermine an individual’s capacity to learn and effectively demonstrate appropriate social skills. Problem behaviors can adversely affect overall social functioning and the quality of interpersonal relationships. Academic competence consists of skills, attitudes, and behaviors that promote a student’s success in school. In addition to subject-specific abilities like reading, writing, and math, it also includes factors like motivation, engagement, and effective study habits. Shaped by both academic, cognitive and psychosocial elements like beliefs and attitudes these skills enable students to perform well and adapt to various academic demands [14].

The social skills domain includes seven subdomains: communication, cooperation, assertion, responsibility, empathy, engagement, and self-control. The problem behaviors domain includes externalizing, bullying, hyperactivity and inattention, internalizing, and autism spectrum. Finally, the academic competence domain includes reading competence, math achievement, and motivation to learn.

A multi-informant tool (teacher, parent, student), the SSIS-RS has (a) two versions of the teacher form, one for preschool students (ages 3–5) and one for elementary/secondary students (ages 5–18); (b) one parent form; and (c) two versions of the student form, one for elementary students (ages 8–12) and one for secondary students (ages 13–18). Only the teacher form for elementary or secondary students contains an academic competence scale.

For the social skills subscales, item-total correlations and factor loadings were examined concurrently. This analysis was performed separately for each rating form (i.e., teacher, parent, and student). Although the proposed seven-factor model for social skills did not fit the data, it was retained in the original study and is widely used in literature [14]. For the problem behaviors subscales, multiple items were assigned to more than one subscale. For example, there is some item overlap between the externalizing and bullying subscales and between the externalizing subscale and the hyperactivity subscale [14]. In addition, the autism spectrum subscale includes reversely coded social skills items [14]. For each subscale, item-total correlation and internal consistency were generated and inspected concurrently. Items were retained for a subscale if the item-total correlation was above .4. However, if the content of an item was conceived to be of high importance and the low item-total correlation was the result of extremely low endorsement rates, or if dropping the item made the internal consistency of the subscale fall below .7, the item was retained.

More recently, and in response to the increasing awareness of social emotional learning (SEL), Gresham and Elliot [28] reexamined the dataset from the 2006 U.S. Census. Their aim was to refine the SSIS-SR to reflect core components of SEL more accurately (e.g., self-awareness, self-management, social awareness, relationship skills, and responsible decision-making), improve the instrument’s alignment with contemporary SEL frameworks, and ensure its continued relevance in assessing social-emotional competencies. While the results of these modifications were promising, the psychometric properties in the updates have not been found to be consistent. For example, seeking to replicate Gresham and Elliot [28], Anthony et al. [29], using a primarily white sample (67%), found that polytomous item response theory revealed 19 and 11 poorly functioning items from the social skills and problem behaviors domains, respectively, in the SSIS teacher form. The use of different analysis methods or the characteristics of the sample in Anthony et al. might have caused discrepancies in the psychometric results, highlighting the importance of applying appropriate cutting-edge analyses and accumulating empirical evidence to validate a measure for a specific population.

Moreover, unlike the extensive research conducted on the translations of the original SSRS, little attention has been given to exploring the factor structure of the updated SSIS-RS in diverse populations. Given the widespread use of the instrument, this gap is significant as it raises concerns about the conceptual equivalence of the assessment across informants from different cultural backgrounds—a question of construct validity [30]. For example, in a Chinese sample, findings supported the proposed SSIS-RS seven-factor structure [8]. In another primarily white sample (68.8%), researchers used an exploratory graph analysis and bi-factor (S-I) modeling to support a four-factor structure in the student self-report measure [31]. Based on the findings of these studies and research preceding them, Panayiotou and colleagues [31] recommended that future SSIS-RS researchers explore the factor structure.

Considering its continuing use, examining the psychometric properties of the SSIS-RS, particularly its construct validity, across diverse populations is essential. The present study, therefore, explored the factor structure of the SSIS-RS, as used by parents and teachers, with a sample of Mexican American preschoolers. Our impetus for this examination was driven by the possibility that Latinx SSIS-RS respondents, as a function of cultural values, held behavioral expectations for children that depart in meaningful ways from those in the standardization samples and that these differences may have result in biased ratings of Latinx children’s social skills [32].

## Methods

### Participants

A total of 761 children were evaluated by their parents and teachers using the SSIS-RS. Forms were collected as part of a more extensive efficacy study exploring the effects of a shared book reading intervention on oral language competence. In the present study, 624 Mexican-ethnicity parents completed the Spanish protocol of the SSIS-RS for 624 children, and 135 teachers completed 755 forms of the English protocol for 755 children. Out of the 761 total children, 618 were commonly assessed by both teachers and parents. Importantly for this study, parent and teacher data were analyzed separately, resulting in *N* = 624 for the parent data and *N* = 755 for the teacher data.

Data were collected from nine schools in two diverse, primarily Latinx, school districts in a South Texas border region. School District A had 24,024 students, with 88% considered at risk, 56% enrolled in bilingual or English Language Learning (ELL) programs, 99% Hispanic, 93% economically disadvantaged, 58% limited English proficient (LEP), and with 97% of the teachers being Hispanic. School District B had 29,242 students with 80% considered at risk of dropping out of school, 49% enrolled in bilingual or ELL, 99% Hispanic, 92% economically disadvantaged, 43% LEP, and with 95% of the teachers being Hispanic.

To provide cultural and linguistic sensitivity, informed consent was obtained from all participants by Latinx graduate students, who spoke Spanish and English. The forms were available in Spanish and English to ensure that all participants understood the study’s purpose, procedures, risks, and benefits, and that they had the opportunity to ask questions. Participants were allowed to complete either the Spanish or the English version. They were told their participation was voluntary and they could withdraw without consequences. The university’s Institutional Review Board (IRB) approved the study.

The ages of the 624 Mexican American children for whom the parent form was completed ranged from 47 to 77 months (*M* = 61.72 months, *SD* = 4.80 months). In terms of gender, 51.4% reported their gender as female, 47.9% as male, and 0.6% was missing gender information. Most parents (81.6%) reported a family income of less than $15,000 (*N* = 294, 47.1%). Among parents who reported mothers’ educational attainment (*N* = 538, 86.2%), the majority had a 9^th^- to 11^th^-grade education (*N* = 155, 24.8%) or reported being high school graduates or having completed a GED (*N* = 168, 26.9%). Similar to the mothers, most of the participating fathers’ educational attainment (*N* = 494, 79.2%) was reported as 9^th^ to 11^th^ grade (*N* = 151, 24.2%) or high school graduation or GED (*N* = 157, 25.2%).

The age of the 755 children for whom the teacher forms of the English version of the SSIS-RS were completed ranged from 51 to 77 months (*M* = 61.67 months, *SD* = 4.60 months). In terms of gender, 50.9% reported their gender as female, 48.5% as male, and 0.7% had missing gender information. The teacher-report SSIS rating scales were completed by 135 teachers (94.8% female and 5.2% male). Regarding teachers’ educational attainment, 125 held a bachelor’s degree and 10 had a master’s degree. Their average number of years of teaching experience was 9.36 years (*SD* = 8.20 years). The average experience teaching pre-kindergarten was 4.93 years (*SD* = 5.14 years).

### Instrument

#### SSIS-RS.

The SSIS-RS includes English versions of the teacher, parent, and student forms as well as Spanish versions of the parent and student forms. In the current study, parents filled out the Spanish SSIS-RS parent form whereas teachers filled out the English version of the teacher form. The Spanish forms were developed and standardized concurrently with the English versions.

Item analysis has been performed to establish psychometric equivalence between English and Spanish versions of the instrument, comparing item-total correlations and internal consistency reliability across subscales [33]. Results revealed similar patterns of correlation magnitudes and rank orders, as well as comparable reliability estimates between versions. These findings provide evidence for the Spanish translation’s structural integrity and construct validity, ensuring its utility for cross-linguistic comparisons [33].

Parents/teachers were asked to rate a child’s behavior on the frequency of each social skill and problem behavior on a 4-point Likert scale (0: *Never* to 3: *Almost always*). With complete data, a social skills scale score may be computed as the sum of the scores on the 46 social skills items for both the teacher and the parent form. In the present study, Cronbach’s alpha for social skills was .95 for the parent report and .97 for the teacher report. The number of items on the problem behaviors scale (excluding the reversely coded social skills items on the autism spectrum subscale) is different across forms: 30 items on the teacher form and 33 items on the parent form. With complete data, a problem behaviors scale score may be computed as the sum of the scores on all problem behavior items. In the present study, Cronbach’s alpha for problem behaviors was .93 and .94 for the parent and the teacher report, respectively.

### Analytical strategy

We first estimated the means, standard deviations, skewness, and kurtosis of all items included in the analyses. Given that the items had only four response categories, a series of ordered-categorical confirmatory factor analysis (CFA) models were then fitted using Mplus software 8.3 [34]. Four separate analyses for the different raters (parents and teachers) and for the different subscales (social skills and problem behaviors) were conducted. (Separate analyses were conducted for parents’ and teachers’ responses to examine the similarities and discrepancies between the raters.)

To account for the nesting of students within classrooms, TYPE = COMPLEX in Mplus was used. Further, to obtain conventional model fit statistics, we utilized robust unweighted least-squares estimation (ESTIMATOR = ULSMV in Mplus), (a) with pairwise deletion (default of ULSMV in Mplus for ordered-categorical data) and (b) with listwise deletion. We examined the following model fit indices to determine the adequacy of the models: The comparative fit index (CFI) [35]; the Tucker-Lewis index (TLI) [36]; the root-mean-square error of approximation (RMSEA) [37,38]; and the standardized root-mean-square residual (SRMR) [39].

Residuals for correlations and modification indices were also examined. In addition, we replicated the results using the robust maximum likelihood estimation (ESTIMATOR = MLR in Mplus) with the probit link. This accounted for missing data using full information maximum likelihood estimation (FIML) [40], but did not provide conventional model fit statistics for our models due to the large number of ordered-categorical items in the models. In total, three different combinations of estimator and missing data handling method were used: ULSMV-pairwise, ULSMV-listwise, and MLR-FIML. We relied on the first two methods to evaluate overall model fit since MLR-FIML did not provide conventional fit statistics. For the best-fitting model that we chose, we interpreted estimates from the MLR-FIML solution since it deals with missing data more appropriately [40].

Given the typical use of the SSIS-RS measure in practice (i.e., one social skills score and one problem behaviors score) for both the parent-report and the teacher-report data, we first fitted a single-factor ordered-categorical CFA to all the social skills items and to all the problem behaviors items separately. If the fit of the single-factor CFA was not acceptable and local fit information indicated underestimation of correlation among items on the same subscales, we proceeded with bi-factor models with one general factor on which all items loaded to capture commonality of all items and multiple domain-specific factors (one for each subscale) and additional commonality among items on the same subscale, where all factors are uncorrelated [41]. However, such models are often under-identified, and often one or more domain-specific factors may have to be dropped from the model (e.g., [41,42]). In such cases, the domain-specific factors, which are still orthogonal to the general factor, may be correlated; such correlations represent partial correlations corrected for common influences of the general factor [42]. We allowed all retained domain-specific factors to correlate with each other (but not the general factor) if modification indices suggested adding such correlations to the model. Finally, we calculated the explained common variance (ECV), which is the proportion of common variance explained by the general factor. ECV is an index of the degree of unidimensionality that is directly related to the relative strength of the general factor compared to the domain-specific factors [43].

## Results

### Descriptive statistics

The mean, standard deviation, skewness, kurtosis, and missing data rate for the social skills and problem behavior items in both the parent-report and teacher-report SSIS-RS rating scales are available in Supplementary Material 1 (S1 Fig) and Supplementary Material 2 (S2 Fig). For problem behaviors, social skills items displayed less obvious non-normal distribution properties. For parent-report social skills items, skewness ranged from −1.26 to .22 and kurtosis ranged from −.99 to .89; for teacher-report social skills items, skewness ranged from −.82 to .53 and kurtosis ranged from −.77 to .43. All parent-report problem behaviors items except for Item 51 (skewness = −.04 and kurtosis = −1.20) were positively skewed, with skewness from .34 to 3.36 and kurtosis from −.74 to 12.27. Similarly, all teacher-report problem behaviors items were positively skewed, with skewness from .74 to 4.32 and kurtosis from −.51 to 22.02. For 10 out of 33 problem behaviors items in the parent report and 20 out of 30 problem behaviors items in the teacher report, more than 70% of the observed data fell in one response category. The missing data rate for each item in the parent-report SSIS rating scales was between .0% and 12.7%. The missing data rate for each item in the teacher SSIS rating scales was between .10% and 2.5%.

### Guide for reading CFA results

The CFA results are reported in the order of parent report social skills, parent report problem behaviors, teacher report social skills, and teacher report problem behaviors. For each form, multiple models were fitted to the data starting from a single-factor model. If model fit was not satisfactory, then, more complicated models such as bi-factor models were fitted followed by modifications to the bi-factor models. For each model fit, three different estimators plus missing data handling combinations were used: ULSMV+pairwise deletion, ULSMV+listwise deletion, and MLR+FIML. Final model estimates are given in Tables 1–4. Readers who are interested in the results for all models can find them in the Supplementary Materials. Readers who are less interested in the technical details can jump to the Summary section which summarizes the results.

**Table 1:** Unstandardized (standardized) factor loadings, factor covariances (correlations), and factor variances in the incomplete bi-factor model with domain-specific factors communication and responsibility dropped and domain-specific factors allowed to correlate fitted to parent-report social skills items (Model SSP4).

Item	General factor				Domain-specific factors				Item *R*^2^ (based on MLR solution)	I-ECV ^d^
	MLR	ULSMV with pairwise deletion		ULSMV with listwise deletion	MLR		ULSMV with pairwise deletion	ULSMV with listwise deletion		
Factor loadings										
					Communication					
4	.60^***^ (.51)^a^	.59^***^ (.52)		.66^***^ (.59)	_		_	_	.26	1.00
10	.88^***^ (.66)	.78^***^ (.63)		.75^***^ (.63)	_		_	_	.43	1.00
14	.91^***^ (.67)	.80^***^ (.64)		.69^***^ (.60)	_		_	_	.45	1.00
20	.31^***^ (.30)	.34^***^ (.33)		.33^***^ (.34)	_		_	_	.09	1.00
24	.82^***^ (.63)	.79^***^ (.63)		.76^***^ (.64)	_		_	_	.40	1.00
30	1.00 (.71)	1.00 (.72)		1.00 (.74)	_		_	_	.50	1.00
40	.70^***^ (.57)	.71^***^ (.59)		.62^***^ (.56)	_		_	_	.33	1.00
					Cooperation					
2	1.32^***^(.73)	1.32^***^ (.72)		1.07^***^ (.71)	1.00 (.39)		1.00 (.44)	1.00 (.37)	.69	.77
7	1.30^***^ (.74)	1.13^***^ (.73)		1.10^***^ (.74)	.89^*^ (.36)		.51^***^ (.26)	.76^***^ (.29)	.68	.81
12	1.02^***^ (.70)	.93^***^ (.69)		.89^***^ (.70)	.31^b^ (.15)		.10^b^ (.06)	.15^b^ (.06)	.52	.94
17	1.43^***^ (.78)	1.29^***^ (.77)		1.22^***^ (.77)	.75^**^ (.29)		.57^***^ (.28)	.85^***^ (.30)	.70	.87
27	.84^***^ (.64)	.77^***^ (.62)		.73^***^ (.61)	.11^b^ (.06)		.19^*^ (.12)	.36^**^ (.17)	.41	1.00
37	1.13^***^ (.75)	.99^***^ (.72)		.87^***^ (.69)	_^c^		_^c^	_^c^	.56	1.00
					Assertion					
1	.15^*^ (.14)	.15^**^ (.15)		.15^**^ (.15)	.75^***^ (.33)		.73^***^ (.32)	.54^***^ (.28)	.13	.15
5	.35^***^ (.32)	.34^***^ (.32)		.32^***^ (.31)	.58^***^ (.25)		.59^***^ (.26)	.60^***^ (.29)	.17	.59
11	.66^***^ (.53)	.62^***^ (.52)		.62^***^ (.54)	.74^***^ (.28)		.71^***^ (.27)	.68^***^ (.30)	.36	.78
15	.71^***^ (.47)	.68^***^ (.48)		.69^***^ (.51)	1.81^***^ (.57)		1.65^***^ (.54)	1.45 ^***^ (.54)	.55	.40
25	.57^***^ (.45)	.56^***^ (.45)		.55^***^ (.47)	1.12^***^ (.42)		1.13^***^ (.42)	.96^***^ (.41)	.38	.53
35	.84^***^ (.64)	.79^***^ (.63)		.68^***^ (.60)	.19^b^ (.07)		.13^b^ (.05)	.11^b^ (.05)	.42	.98
45	.35^***^ (.30)	.33^***^ (.29)		.37^***^ (.33)	1.00 (.41)		1.00 (.41)	1.00 (.45)	.26	.35
					Responsibility					
6	.91^***^ (.67)	.83^***^ (.65)		.82^***^ (.67)	_		_	_	.45	1.00
16	.87^***^ (.65)	.77^***^ (.62)		.71^***^ (.61)	_		_	_	.43	1.00
22	1.15^***^ (.75)	.99^***^ (.72)		.93^***^ (.72)	_		_	_	.57	1.00
26	1.19^***^ (.76)	1.06^***^ (.74)		1.01^***^ (.74)	_		_	_	.58	1.00
32	1.13^***^ (.75)	1.00^***^ (.72)		.94^***^ (.72)	_		_	_	.56	1.00
42	1.08^***^ (.73)	.93^***^ (.69)		.84^***^ (.68)	_		_	_	.53	1.00
					Empathy					
3	.73^***^ (.54)	.66^***^ (.53)		.56^***^ (.50)	.78^***^ (.40)		.79^***^ (.35)	.71^***^ (.29)	.45	.64
8	.97^***^ (.65)	.90^***^ (.64)		.82^***^ (.63)	.75^***^ (.36)		.86^***^ (.34)	.90^***^ (.32)	.55	.76
13	.74^***^ (.58)	.71^***^ (.57)		.60^***^ (.53)	.46^*^ (.25)		.61^***^ (.27)	.73^***^ (.29)	.40	.85
18	1.10^***^ (.67)	.97^***^ (.66)		.92^***^ (.67)	1.00 (.43)		1.00 (.37)	1.00 (.33)	.63	.71
28	.81^***^ (.53)	.79^***^ (.53)		.74^***^ (.51)	1.12^***^ (.52)		1.50^***^ (.55)	1.94^***^ (.60)	.56	.50
38	1.08^***^ (.66)	1.00^***^ (.66)		.90^***^ (.63)	.95^***^ (.42)		1.11^***^(.40)	1.45^***^ (.46)	.61	.72
					Engagement					
9	.69^***^ (.55)	.67^***^ (.55)		.67^***^ (.57)	.33^***^ (.27)		.39^***^ (.27)	.40^***^ (.28)	.37	.81
19	.80^***^ (.50)	.75^***^ (.50)		.76^***^ (.53)	.95^***^ (.60)		1.01^***^ (.57)	1.01^***^ (.57)	.61	.41
23	.76^***^ (.47)	.69^***^ (.47)		.63^***^ (.46)	1.00 (.62)		1.00 (.58)	1.00 (.59)	.61	.36
29	1.16^***^ (.71)	1.03^***^ (.70)		.94^***^ (.69)	.55^***^ (.34)		.51^***^ (.29)	.46^***^ (.27)	.62	.81
33	.67^***^ (.54)	.63^***^ (.53)		.57^***^ (.52)	.28^***^ (.23)		.25^***^ (.18)	.31^***^ (.23)	.34	.85
39	.90^***^ (.61)	.87^***^ (.60)		.79^***^ (.59)	.62^***^ (.42)		.74^***^ (.44)	.74^***^ (.45)	.54	.68
43	.58^***^ (.44)	.58^***^ (.46)		.54^***^ (.44)	.61^***^ (.46)		.65^***^ (.44)	.76^***^ (.50)	.41	.46
					Self-control					
21	.88^***^ (.66)	.81^***^ (.64)		.80^***^ (.66)	.11^b^ (.06)		.15^*^ (.10)	.24^***^ (.15)	.44	1.00
31	.82^***^ (.54)	.76^***^ (.51)		.68^***^ (.49)	1.00 (.51)		1.00 (.56)	1.00 (.56)	.56	.52
34	.84^***^ (.55)	.74^***^ (.53)		.68^***^ (.51)	1.01^***^ (.51)		.78^***^ (.47)	.90^***^ (.52)	.56	.54
36	1.00 ^***^ (.69)	.92^***^ (.67)		.88^***^ (.67)	.36^**^ (.19)		.36^***^ (.22)	.45^***^ (.26)	.52	.92
41	.92^***^ (.60)	.84^***^ (.57)		.82^***^ (.57)	.92^***^ (.46)		.86^***^ (.49)	1.03^***^ (.54)	.57	.63
44	.62^***^ (.50)	.58^***^ (.50)		.54^***^ (.55)	.42^***^ (.26)		.35^***^ (.25)	.48^***^ (.31)	.32	.78
46	.93^***^ (.62)	.83^***^ (.58)		.78^***^ (.57)	.80^***^ (.41)		.80^***^ (.47)	.88^***^ (.49)	.55	.69
Factor variances										
			MLR			ULSMV with pairwise deletion				ULSMV with listwise deletion
General Factor (Social Skills)			.99			1.06				1.21
Communication			_			_				_
Cooperation			.49			.70				.37
Assertion			.22			.23				.30
Responsibility			_			_				_
Empathy			.49			.32				.25
Engagement			1.00			.77				.79
Self-Control			.60			.75				.70
Covariances (correlations) among domain-specific factors										
			MLR			ULSMV with pairwise deletion				ULSMV with listwise deletion
Assertion with Cooperation			−.10^b^ (−.31)^a^			−.18^***^ (−.47)				−.11^**^ (−.34)
Empathy with Cooperation			−.09^b^ (−.19)			−.16^***^ (−.35)				−.14^***^ (−.45)
Empathy with Assertion			.23^***^ (.70)			.17^***^ (.63)				.16^***^ (.58)
Engagement with Cooperation			−.26^*^ (−.37)			−.37^***^ (−.50)				−.25^***^ (−.46)
Engagement with Assertion			.32^***^ (.68)			.28^***^ (.66)				.33^***^ (.68)
Engagement with Empathy			.36^***^ (.51)			.25^***^ (.51)				.23^***^ (.52)
Self-control with Cooperation			−.17^**^ (−.31)			−.17^**^ (−.24)				−.15^**^ (−.30)
Self-control with Assertion			−.15^**^ (−.41)			−.18^***^ (−.43)				−.19^***^ (−.41)
Self-control with Empathy			−.12^*^ (−.21)			−.12^**^ (−.24)				−.09^*^ (−.21)
Self-control with Engagement			−.20^*^ (−.25)			−.21^***^ (−.28)				−.20^***^ (−.27)

*Note*. *N* = 624 (*N* = 483 for ULSMV with listwise deletion). * *p* < .05; ** *p* < .01; *** *p* < .001.

^a^For factor loadings, the completely standardized solution is included in parentheses following the unstandardized factor loadings. For covariances among domain-specific factors, the completely standardized solution (i.e., factor correlations) are included in parentheses following the unstandardized factor covariances.

^b^Factor loading or factor covariance was nonsignificant based on the unstandardized solution.

^c^Factor loading of Item 37 on the domain-specific factor cooperation was constrained to 0.

^d^Item-level explained common variance (I-ECV) was calculated as (% item total variance explained by general factor)/(item *R*^*2*^).

**Table 2:** Unstandardized (standardized) factor loadings and factor variances in the one-factor model fitted to parent-reports on problem behaviors items (autism subscale excluded) (Model PBP1).

Item	MLR		ULSMV with pairwise deletion		ULSMV with listwise deletion		Item *R*^*2*^ (based on MLR solution)
Factor loadings							
47	.37^***^(.48)^a^		.37^***^ (.44)		.36^***^ (.47)		.23
48	.48^***^ (.57)		.48^***^ (.55)		.45^***^ (.55)		.33
49	.50^***^ (.59)		.57^***^ (.61)		.55^***^ (.63)		.35
50	.50^***^ (.59)		.57^***^ (.61)		.51^***^ (.60)		.35
51	.55^***^ (.63)		.52^***^ (.58)		.51^***^ (.60)		.39
52	.57^***^ (.64)		.65^***^ (.66)		.64^***^ (.69)		.41
53	.47^***^ (.57)		.46^***^ (.53)		.47^***^ (.57)		.32
54	.72^***^ (.72)		.69^***^ (.68)		.68^***^ (.71)		.52
55	.66^***^ (.69)		.68^***^ (.68)		.66^***^ (.70)		.48
56	1.00 (.83)		1.00 (.80)		1.00 (.83)		.68
57	.61^***^ (.67)		.68^***^ (.68)		.61^***^ (.67)		.44
58	.77^***^ (.75)		.73^***^ (.70)		.68^***^ (.70)		.56
59	.77^***^ (.75)		.80^***^ (.74)		.80^***^ (.76)		.56
60	.79^***^ (.75)		.80^***^ (.73)		.76^***^ (.75)		.57
61	.48^***^ (.57)		.52^***^ (.57)		.49^***^ (.58)		.33
62	.74^***^ (.74)		.80^***^ (.73)		.74^***^ (.73)		.54
63	.88^***^ (.79)		1.01^***^ (.81)		1.01^***^ (.83)		.62
64	.73^***^ (.73)		.78^***^ (.72)		.74^***^ (.73)		.53
65	.81^***^ (.76)		.77^***^ (.72)		.75^***^ (.74)		.58
66	.79^***^ (.75)		.92^***^ (.78)		.86^***^ (.78)		.57
67	.71^***^ (.72)		.69^***^ (.68)		.67^***^ (.70)		.52
68	.64^***^ (.68)		.70^***^ (.69)		.69^***^ (.71)		.47
69	.35^***^ (.45)		.37^***^ (.45)		.37^***^ (.48)		.20
70	.82^***^ (.77)		.78^***^ (.72)		.74^***^ (.73)		.59
71	.30^***^ (.40)		.36^***^ (.44)		.35^***^ (.46)		.16
72	.56^***^ (.64)		.60^***^ (.63)		.59^***^ (.66)		.40
73	.36^***^ (.46)		.35^***^ (.43)		.31^***^ (.42)		.22
74	.75^***^ (.74)		.87^***^ (.76)		.82^***^ (.77)		.54
75	.65^***^ (.69)		.80^***^ (.73)		.72^***^ (.73)		.48
76	.65^***^ (.69)		.69^***^ (.68)		.64^***^ (.69)		.48
77	.57^***^ (.64)		.65^***^ (.66)		.62^***^ (.67)		.41
78	.81^***^ (.76)		.80^***^ (.73)		.74^***^ (.73)		.58
79	.35^***^ (.46)		.40^***^ (.47)		.38^***^ (.49)		.21
Factor variances							
		MLR		ULSMV with pairwise deletion		ULSMV with listwise deletion	
General Factor (Problem Behaviors)		2.13		1.83		2.15	

*Note*. *N* = 624 (*N* = 548 for ULSMV with listwise deletion). ^*^p < .05; ^**^p < .01; ^***^p < .001.

^a^For factor loadings, the completely standardized solution is included in parentheses following the unstandardized factor loadings.

**Table 3:** Unstandardized (standardized) factor loadings, factor covariances (correlations), and factor variances in the bi-factor model with domain-specific communication and engagement factors dropped and domain-specific factors allowed to correlate fitted to teacher-report social skills items (Model SST4).

Item	General factor			Domain-specific factors				Item *R*^*2*^ (based on MLR solution)	I-ECV^d^
	MLR	ULSMV with pairwise deletion	ULSMV with listwise deletion	MLR		ULSMV with pairwise deletion	ULSMV with listwise deletion		
	Factor loadings								
				Communication					
4	.66^***^(.65)^a^	.67^***^(.65)	.69^***^(.66)	_		_	_	.42	1.00
10	.98^***^(.78)	.86^***^(.74)	.89^***^(.75)	_		_	_	.61	1.00
14	.63^***^(.63)	.57^***^(.59)	.58^***^(.60)	_		_	_	.39	1.00
20	1.00 (.79)	1.00 (.79)	1.00 (.79)	_		_	_	.62	1.00
24	.79^***^(.71)	.79^***^(.71)	.79^***^(.71)	_		_	_	.51	1.00
30	.92^***^(.76)	.85^***^(.74)	.83^***^(.73)	_		_	_	.58	1.00
40	.79^***^(.71)	.76^***^(.69)	.77^***^(.70)	_		_	_	.50	1.00
				Cooperation					
2	.88^***^(.62)	.83^***^(.63)	.88^***^(.64)	.68^***^(.57)		.56^***^(.50)	.57^***^(.50)	.70	.54
7	.75^***^(.46)	.86^***^(.52)	.90^***^(.53)	1.00 (.74)		1.00 (.71)	1.00 (.71)	.77	.27
12	.94^***^(.75)	.86^***^(.72)	.85^***^(.72)	.26^***^(.25)		.22^***^(.22)	.22^***^(.22)	.62	.90
17	1.19^***^(.72)	1.13^***^(.72)	1.14^***^(.72)	.72^***^(.52)		.64^***^(.48)	.63^***^(.48)	.78	.67
27	.58^***^(.52)	.66^***^(.56)	.68^***^(.57)	.46^***^(.49)		.49^***^(.49)	.49^***^(.49)	.51	.53
37	1.16^***^(.60)	1.47^***^(.65)	1.52^***^(.66)	1.11^***^(.69)		1.32^***^(.68)	1.27^***^(.67)	.84	.43
				Assertion					
1	.27^***^(.31)	.25^***^(.29)	.23^***^(.27)	.35^*^(.35)		.31^***^(.36)	.32^***^(.34)	.22	.45
5	.33^***^(.31)	.27^***^(.26)	27^***^(.26)	.73^*^(.60)		.58^***^(.58)	.65^***^(.59)	.46	.22
11	.77^***^(.55)	.71^***^(.48)	.68^***^(.49)	1.00 (.63)		1.00 (.69)	1.00 (.66)	.69	.43
15	.69^***^(.62)	.58^***^(.55)	.56^***^(.54)	.46^***^(.36)		.41^***^(.39)	.44^***^(.39)	.51	.75
25	.82^***^(.67)	.76^***^(.65)	.77^***^(.65)	.55^b^(.39)		.39^***^(.34)	.47^***^(.37)	.60	.75
35	1.09^***^(.75)	1.04^***^(.75)	1.06^***^(.75)	.61^b^(.37)		.48^***^(.36)	.56^***^(.37)	.71	.79
45	.77^***^(.68)	.74^***^(.67)	.78^***^(.68)	.36^b^(.27)		.26^***^(.24)	.34^***^(.27)	.53	.87
				Responsibility					
6	.64^***^(.47)	.71^***^(.53)	.72^***^(.54)	1.12^***^(.68)		1.04^***^(.62)	1.04^***^(.60)	.68	.32
16	1.12^***^(.77)	1.13^***^(.78)	1.15^***^(.78)	.62^***^(.36)		.56^***^(.31)	.59^***^(.31)	.71	.83
22	1.29^***^(.72)	1.34^***^(.73)	1.40^***^(.74)	1.15^***^(.54)		1.21^***^(.53)	1.32^***^(.54)	.81	.64
26	.83^***^(.63)	.85^***^(.65)	.85^***^(.66)	.79^***^(.51)		.75^***^(.46)	.73^***^(.44)	.65	.62
32	1.03^***^(.67)	1.05^***^(.68)	1.04^***^(.69)	1.00 (.55)		1.00 (.52)	1.00 (.51)	.74	.61
42	.93^***^(.77)	.90^***^(.75)	.92^***^(.76)	_^c^		_^c^	_^c^	.59	1.00
				Empathy					
3	.82^***^(.65)	.82^***^(.63)	.88^***^(.64)	.54^**^(.45)		.75^***^(.49)	.96^***^(.53)	.62	.68
8	.66^***^(.64)	.69^***^(.66)	.69^***^(.66)	.14^b^(.15)		.06^***^(.05)	−.05^b^(−.03)	.43	.95
13	.96^***^(.69)	1.01^***^(.67)	1.03^***^(.67)	.61^**^(.46)		.93^***^(.53)	1.09^***^(.54)	.69	.69
18	1.59^***^(.77)	1.43^***^(.77)	1.38^***^(.79)	1.00 (.51)		1.00 (.47)	1.00 (.43)	.86	.68
28	1.18^***^(.77)	1.11^***^(.77)	1.10^***^(.77)	.55^***^(.38)		.56^***^(.34)	.59^***^(.31)	.74	.80
38	1.29^***^(.76)	1.23^***^(.76)	1.19^***^(.77)	.75^***^(.46)		.79^***^(.42)	.79^***^(.39)	.79	.73
				Engagement					
9	1.05^***^(.80)	.88^***^(.75)	.86^***^(.74)	_		_	_	.64	1.00
19	1.02^***^(.79)	1.02^***^(.79)	.98^***^(.78)	_		_	_	.63	1.00
23	1.01^***^(.79)	.90^***^(.75)	.88^***^(.74)	_		_	_	.63	1.00
29	1.14^***^(.83)	.96^***^(.77)	.95^***^(.77)	_		_	_	.68	1.00
33	1.10^***^(.82)	.91^***^(.76)	.87^***^(.74)	_		_	_	.66	1.00
39	.94^***^(.77)	.74^***^(.68)	.72^***^(.68)	_		_	_	.59	1.00
43	.77^***^(.70)	.67^***^(.65)	.67^***^(.65)	_		_	_	.49	1.00
				Self-control					
21	.58^***^(.49)	.82^***^(.49)	.95^***^(.51)	.67^***^(.57)		1.69^***^(.73)	2.14^***^(.75)	.56	.43
31	.72^***^(.54)	.68^***^(.56)	.67^***^(.58)	.80^***^(.60)		.85^***^(.50)	.79^***^(.45)	.65	.45
34	.99^***^(.73)	.89^***^(.72)	.89^***^(.74)	.52^***^(.38)		.47^***^(.27)	.38^***^(.21)	.67	.79
36	.88^***^(.68)	.80^***^(.68)	.83^***^(.70)	.57^***^(.43)		.50^***^(.30)	.43^***^(.24)	.64	.72
41	.67^***^(.62)	.65^***^(.61)	.67^***^(.62)	.34^**^(.31)		.47^***^(.31)	.50^***^(.30)	.48	.79
44	.70^***^(.66)	.70^***^(.66)	.71^***^(.67)	.17^b^(.16)		−.01^b^(−.01)	−.11^b^(−.07)	.46	.96
46	.87^***^(.57)	.78^***^(.59)	.79^***^(.61)	1.00 (.65)		1.00 (.54)	1.00 (.51)	.74	.43
Factor variances									
			MLR		ULSMV with pairwise deletion		ULSMV with listwise deletion		
General Factor (Social Skills)			1.64		1.61		1.62		
Communication			_		_		_		
Cooperation			2.36		2.23		2.35		
Assertion			1.26		1.69		1.39		
Responsibility			1.16		1.05		.96		
Empathy			1.83		1.20		.93		
Engagement			_		_		_		
Self-Control			1.63		.83		.71		
Factor covariances (correlations) among domain-specific factors									
			MLR		ULSMV with pairwise deletion		ULSMV with listwise deletion		
Assertion with Cooperation			−.81^b^(−.47)^a^		−.94^***^(−.48)		−.87^***^(−.48)		
Responsibility with Cooperation			1.53^***^(.92)		1.42^***^(.93)		1.38^***^(.92)		
Responsibility with Assertion			−.66^b^(−.55)		−.74^***^(−.56)		−.67^***^(−.58)		
Empathy with Cooperation			.04^b^(.02)		−.1^b^(−.07)		−.14^b^(−.09)		
Empathy with Assertion			.22^b^(.15)		.27^*^(.19)		.21^*^(.19)		
Empathy with Responsibility			.17^b^(.12)		.04^b^(.04)		−.02^b^(−.02)		
Self-control with Cooperation			.70^***^(.36)		.48^***^(.36)		.42^***^(.33)		
Self-control with Assertion			−.24^b^(−.17)		−.32^*^(−.27)		−.31^**^(−.31)		
Self-control with Responsibility			.82^***^(.60)		.56^***^(.60)		.49^***^(.59)		
Self-control with Empathy			.35^b^(.20)		.10^b^(.10)		.01^b^(.02)		

*Note*. *N* = 754 (*N* = 680 for ULSMV with listwise deletion). * *p* < .05; ** *p* < .01; *** *p* < .001.

a For factor loadings, the completely standardized solution is included in parentheses following the unstandardized factor loadings. For covariances among domain-specific factors, the completely standardized solution (i.e., factor correlations) are included in parentheses following the unstandardized factor covariances.

b Factor loading or factor covariance was nonsignificant based on the unstandardized solution.

c Factor loading of Item 42 on the domain-specific factor responsibility was constrained to 0.

d Item-level explained common variance (I-ECV) was calculated as (% item total variance explained by general factor)/(item *R*^*2*^).

**Table 4:** Unstandardized (standardized) factor loadings and factor variances in the incomplete bi-factor model with specific factor for internalizing fitted to teacher-report problem behaviors items (Model PBT2).

Item	General factor				Domain-specific factor				Item *R*^2^ (based on MLR solution)	I-ECV^c^
	MLR	ULSMV with pairwise deletion	ULSMV with listwise deletion		MLR	ULSMV with pairwise deletion		ULSMV with listwise deletion		
Factor loadings										
					Internalizing problem					
56^b^	.72^***^(.70)^a^	.63^***^(.67)	.63^***^(.66)		.72^***^(.56)	.60^***^(.49)		.61^***^(.49)	.76	.64
62	.18^***^(.26)	.12^**^(.18)	.14^***^(.19)		.57^***^(.62)	.55^***^(.61)		.55^***^(.61)	.45	.15
64	.47^***^(.46)	.46^***^(.44)	.46^***^(.45)		1.00 (.73)	1.00 (.74)		1.00 (.73)	.75	.28
68	.57^***^(.71)	.56^***^(.70)	.56^***^(.70)		.32^***^(.30)	.26^**^(.25)		.26^**^(.25)	.60	.84
70	.38^***^(.51)	.31^***^(.43)	.32^***^(.44)		.53^***^(.53)	.53^***^(.55)		.51^***^(.53)	.53	.49
74	.64^***^(.62)	.60^***^(.58)	.60^***^(.58)		.86^***^(.62)	.84^***^(.63)		.88^***^(.64)	.76	.51
76	.74^***^(.82)	.72^***^(.80)	.77^***^(.81)		.19^*^(.16)	.14^*^(.12)		.16^***^(.13)	.69	.97
47^b^	.57^***^(.75)	.58^***^(.74)	.58^***^(.74)						.56	1.00
48	.37^***^(.59)	.37^***^(.57)	.38^***^(.58)						.35	1.00
49^b^	.86^***^(.86)	.78^***^(.83)	.78^***^(.83)						.74	1.00
50	.53^***^(.73)	.58^***^(.74)	.59^***^(.74)						.53	1.00
51^b^	.74^***^(.83)	.69^***^(.80)	.70^***^(.79)						.68	1.00
52	.77^***^(.84)	.75^***^(.82)	.76^***^(.82)						.70	1.00
53^b^	.65^***^(.79)	.61^***^(.76)	.61^***^(.75)						.62	1.00
54	.67^***^(.80)	.73^***^(.81)	.80^***^(.83)						.64	1.00
55^b^	1.00 (.89)	1.00 (.89)	1.00 (.88)						.80	1.00
57^b^	.70^***^(.81)	.71^***^(.80)	.73^***^(.81)						.66	1.00
58	.58^***^(.75)	.62^***^(.76)	.65^***^(.77)						.57	1.00
59	.86^***^(.86)	.90^***^(.86)	.93^***^(.87)						.74	1.00
60	.50^***^(.71)	.56^***^(.73)	.58^***^(.74)						.50	1.00
61^b^	.94^***^ (.88)	1.00^***^ (.88)	1.00^***^ (.88)						.77	1.00
63	.63^***^(.78)	.67^***^(.78)	.68^***^(.79)						.61	1.00
65	.55^***^(.73)	.53^***^(.71)	.54^***^(.71)						.54	1.00
66	.67^***^(.80)	.79^***^(.83)	.81^***^(.83)						.64	1.00
67	.95^***^(.88)	.84^***^(.85)	.84^***^(.84)						.78	1.00
69	.97^***^(.89)	.88^***^(.86)	.89^***^(.86)						.79	1.00
71	.61^***^(.77)	.55^***^(.72)	.55^***^(.72)						.60	1.00
72	.57^***^(.75)	.64^***^(.77)	.65^***^(.77)						.56	1.00
73	.88^***^(.87)	.90^***^(.86)	.89^***^(.86)						.75	1.00
75	.68^***^(.80)	.66^***^(.78)	.70^***^(.79)						.65	1.00
Factor variances										
				MLR			ULSMV with pairwise deletion		ULSMV with listwise deletion	
General Factor (Problem Behavior)				3.91			3.60		3.51	
Internalizing problems				2.15			2.12		2.02	

*Note*. *N* = 754 (*N* = 720 for ULSMV with listwise deletion). * *p* < .05; ** *p* < .01; *** *p* < .001.

^a^For factor loadings, the completely standardized solution is included in parentheses following the unstandardized factor loadings.

^b^Items appeared in more than one subscale.

^c^Item-level explained common variance (I-ECV) was calculated as (% item total variance explained by general factor)/(item *R*^*2*^).

#### Parent report: Social skills.

S1–S4 Figs depict the CFA models fitted for the parent-reported social skills. As illustrated, we first fitted a single-factor CFA to all the parent-report items on the social skills subscales (Model SSP1; a total of 46 items). The model fit based on ULSMV estimation was not acceptable with the default pairwise deletion [$\chi^{2}(989)=\text{2}5\text{8}6.\text{8}3\text{,}p<.\text{0}0\text{1}$; RMSEA = .05; CFI = .91; TLI = .91; SRMR = .07], particularly with listwise deletion [$\chi^{2}(989)=\text{2}4\text{0}9.\text{2}0\text{,}p<.\text{0}0\text{1}$; RMSEA = .06; CFI = .89; TLI = .89; SRMR = .08]. Examination of the residuals for correlations revealed a trend of positive residuals for correlations with a magnitude exceeding .20 (sometimes .30) for items on the same subscales, indicating that the model tended to underestimate correlations among items on the same subscales. Next, we fitted a correlated seven-factor model, which did not converge to a proper solution using ULSMV with pairwise deletion or MLR, likely due to very high correlations among some of the factors representing the subscales (5 out of 21 factor correlations were above .90 with the highest being .97 in the ULSMV solution with listwise deletion).

Subsequently, we fitted a bi-factor model with a general factor on which all items loaded and domain-specific factors for each subscale, where all common factors were uncorrelated (Model SSP2). The model fit of Model SSP2 based on ULSMV estimation was improved compared to the single-factor model (Model SSP1) with the pairwise deletion [$\chi^{2}(\text{9}4\text{3})=\text{2}0\text{4}4.\text{0}3\text{,}p<.\text{0}0\text{1}$; RMSEA = .04; CFI = .94; TLI = .93; SRMR = .06]. Model SSP2 did not converge using listwise deletion.

For a formal statistical model comparison, we conducted an adjusted chi-square difference test using the DIFFTEST option in Mplus [34]. The DIFFTEST revealed that the single-factor model (SSP1) fitted significantly worse than the bi-factor model (SSP2) using pairwise deletion, $\chi^{2}(46)=1018.90,p<\mathrm{.001}$. S4 Fig shows the model solutions from the models using ULSMV estimation with pairwise deletion and MLR estimation, respectively. Negative and/or nonsignificant factor loadings and nonsignificant factor variances for the communication and responsibility factors indicated misspecification of the complete bi-factor model [41,42].

Next, we fitted Model SSP3, in which we dropped two domain-specific factors, communication and responsibility, and constrained the domain-specific factor loading of Item 37 on the domain-specific factor cooperation to zero (this loading was negative and nonsignificant in the MLR solution, but positive and nonsignificant in the ULSMV solutions). The model fit of Model SSP3 based on ULSMV estimation was improved compared to the single-factor model (Model SSP1) with either the default pairwise deletion [$\chi^{2}(\text{9}5\text{7})=\text{2}1\text{0}9.\text{4}7\text{,}p<.\text{0}0\text{1}$; RMSEA = .04; CFI = .94; TLI = .93; SRMR = .06] or listwise deletion [$\chi^{2}(\text{9}5\text{7})=\text{1}9\text{7}9.\text{8}1\text{,}p<.\text{0}0\text{1}$; RMSEA = .05; CFI = .92; TLI = .91; SRMR = .07]. The DIFFTEST results using pairwise deletion revealed that the model with additional restrictions (Model SSP3) fit significantly worse than the model without the restrictions (Model SSP2), $\chi^{2}(14)=158.26,p<\mathrm{.001}$. Although the DIFFTEST suggested better fit of Model SSP2 than Model SSP3, we decided to accept Model SSP3 because it was a more parsimonious model with fit statistics similar to RMSEA, CFI, TLI, and SRMR. Examination of the model solutions did not reveal any sign of an under-identified general or domain-specific factor. The highest modification indices of this model were all associated with covariances among domain-specific factors.

In Model SSP4, our final model for parent reports on social skills items, we allowed the remaining domain-specific factors to correlate on top of the previous model (Model SSP3). Table 1 shows the model solutions from the models using ULSMV estimation with pairwise/listwise deletion and MLR estimation, respectively. The model fit for Model SSP4 based on ULSMV estimation was adequate with either the default pairwise deletion [$\chi^{2}(\text{9}4\text{7})=\text{1}8\text{1}0.\text{0}9\text{,}p<.\text{0}0\text{1}$; RMSEA = .04; CFI = .95; TLI = .95; SRMR = .05] or listwise deletion [$\chi^{2}(\text{9}4\text{7})=\text{1}7\text{3}7.\text{3}9\text{,}p<.\text{0}0\text{1}$; RMSEA = .04; CFI = .94; TLI = .93; SRMR = .06]. The DIFFTEST revealed that Model SSP3 fitted significantly worse than Model SSP4, $\chi^{2}(10)=236.80,p<\mathrm{.001}$ for pairwise deletion, and $\chi^{2}(10)=211.55,p<\mathrm{.001}$ for listwise deletion. In addition to this formal statistical test, RMSEA, CFI, TLI, and SRMR statistics also showed that Model SSP4 was a better fitting model than Model SSP3 for both pairwise and listwise deletion. The standardized factor loadings on the general factor social skills from the three solutions of Model SSP4 were very similar, with discrepancies all in the second decimal place. However, discrepancies in the standardized factor loadings on domain-specific factors were larger across the three solutions for some items on the cooperation and empathy subscales. Discrepancies in the correlations among domain-specific factors were larger across the three solutions, with the greatest discrepancy (.26) occurring for the correlation between the empathy and cooperation domain-specific factors, likely due to the influence of missing data.

For interpretation, we focus on the parameter estimates in the MLR solution, which corrects for the nesting structure and accounts for missing data. Based on the MLR solution of Model SSP4, the ECV was .78, meaning that the general factor social skills explained 78% of the common variance extracted from the parent-report social skills items, with 22% of the common variance spread across the retained domain-specific factors. This indicates a strong general factor.

In terms of correlations among domain-specific factors (representing partial correlations corrected for common influences of the general factor), the self-control domain-specific factor was negatively and significantly correlated with the domain-specific factors cooperation, assertion, empathy, and engagement. The engagement domain-specific factor was negatively and significantly correlated with cooperation but not significantly correlated with assertion or empathy. Finally, the domain-specific factors empathy, assertion, and engagement were positively and significantly correlated with each other (see Table 1 for more details).

#### Parent report: Problem behaviors.

S5 Fig depicts the CFA models fitted for the parent-reported problem behaviors. As illustrated, we fitted a single-factor CFA to all the parent-report items on the problem behaviors scale, excluding the reversely coded social skills items on the autism subscale (Model PBP1; a total of 33 items). The model fit based on ULSMV estimation was adequate with either the default pairwise deletion [$\chi^{2}(\text{4}9\text{5})=\text{1}1\text{5}7.\text{2}3\text{,}p<.\text{0}0\text{1}$; RMSEA = .05; CFI = .94; TLI = .93; SRMR = .07] or listwise deletion [$\chi^{2}(495)=\text{1}0\text{5}1.\text{8}9\text{,}p<.\text{0}0\text{1}$; RMSEA = .05; CFI = .93; TLI = .93; SRMR = .07]. Given the relatively small issue of underestimating correlations among items on the same subscales and considering the complexity of fitting a complete bi-factor model based on the subscale structure with cross-loading items, we decided to treat Model PBP1 as our final model for parent reports on problem behaviors items.

Table 2 shows the model solutions for Model PBP1 using ULSMV estimation with pairwise/listwise deletion and MLR estimation, respectively. The standardized factor loadings from the three solutions of Model PBP1 were very similar, with discrepancies in the second or third decimal place. For interpretation, we focus on the parameter estimates in the MLR solution, which corrects for the nesting structure and accounts for missing data. As seen in Table 2, most problem behaviors items were rather strong indicators of the problem behaviors factor, with standardized factor loadings ranging between .40 and .83. Of the 33 items, 14 (42.4%) had item *R*^2^ above .50.

#### Teacher report: Social skills.

S6–S9 Figs depict the CFA models fitted for the teacher-reported social skills. We first fitted a single-factor CFA to all the teacher-report items on the social skills subscales (Model SST1; a total of 46 items). The model fit based on ULSMV estimation was not acceptable with either the default pairwise deletion [$\chi^{2}(989)=\text{3}4\text{7}5.\text{2}5\text{,}p<.\text{0}0\text{1}$; RMSEA = .06; CFI = .87; TLI = .86; SRMR = .09] or listwise deletion [$\chi^{2}(989)=\text{3}1\text{8}7.\text{6}0\text{,}p<.\text{0}0\text{1}$; RMSEA = .06; CFI = .88; TLI = .87; SRMR = .09]. Examination of the residuals for correlations (the RESIDUAL output in Mplus when the delta parameterization was used for ordered-categorical CFA) revealed that the model tended to underestimate the correlations among items on the same subscales.

Therefore, we fitted a bi-factor model with a general factor on which all items loaded and domain-specific factors for each subscale, where all common factors were uncorrelated (Model SST2). The model fit of Model SST2 based on ULSMV estimation was improved compared to the single-factor model, with pairwise deletion [$\chi^{2}(\text{9}4\text{3})=\text{2}7\text{5}8.\text{4}5\text{,}p<.\text{0}0\text{1}$; RMSEA = .05; CFI = .90; TLI = .89; SRMR = .08] and listwise deletion [$\chi^{2}(\text{9}4\text{3})=\text{2}5\text{4}8.\text{7}8\text{,}p<.\text{0}0\text{1}$; RMSEA = .05; CFI = .91; TLI = .90; SRMR = .08]. The DIFFTEST revealed that Model SST1 fitted significantly worse than Model SST2, $\chi^{2}(46)=1332.80,p<\mathrm{.001}$ for pairwise deletion, and $\chi^{2}(46)=1189.58,p<\mathrm{.001}$ for listwise deletion. S5 Fig contains the model solutions from the models using ULSMV estimation with pairwise/listwise deletion and MLR estimation, respectively. Negative and/or nonsignificant factor loadings and nonsignificant factor variances for the communication and engagement factors suggest these factors may not exist as domain-specific factors over and above the general factor social skills (i.e., the common variance of the items on the subscale communication and engagement is likely explained by the general factor).

Next, we fitted Model SST3, in which we dropped two domain-specific factors, communication and engagement, and constrained the domain-specific factor loading of Item 42 on the domain-specific factor responsibility to zero (this loading was negative in all three solutions). The model fit of Model SST3 based on ULSMV estimation was improved compared to the single-factor model with either the default pairwise deletion [$\chi^{2}(\text{9}5\text{8})=\text{2}8\text{6}2.\text{2}0\text{,}p<.\text{0}0\text{0}1$; RMSEA = .05; CFI = .90; TLI = .89; SRMR = .08] or listwise deletion [$\chi^{2}(\text{9}5\text{8})=\text{2}6\text{4}3.\text{1}8\text{,}p<.\text{0}0\text{0}1$; RMSEA = .05; CFI = .91; TLI = .90; SRMR = .08]. The DIFFTEST revealed that the model with more restrictions (SST3) fitted significantly worse than the model with fewer restrictions (SST2), $\chi^{2}(15)=269.68,p<\mathrm{.001}$ for pairwise deletion and $\chi^{2}(15)=250.89,p<\mathrm{.001}$ for listwise deletion. However, for both pairwise and listwise deletion cases, the RMSEA, CFI, TLI, and SRMR fit statistics showed similar fit between the two models. Therefore, we chose Model SST3 for its parsimony. Examination of the model solutions did not reveal any sign of under-identified general or domain-specific factor. The highest modification indices of this model were all associated with covariances among domain-specific factors.

In Model SST4, our final model for teacher reports on social skills items, we allowed the remaining domain-specific factors to correlate while keeping the general factor uncorrelated with any domain-specific factor on top of Model SST3. Table 3 shows the model solutions from the models using MLR estimation and ULSMV estimation with pairwise/listwise deletion, respectively. As illustrated, the model fit for Model SST4 based on ULSMV estimation was improved compared to Model SST3, with either the default pairwise deletion [$\chi^{2}(\text{9}4\text{8})=\text{2}2\text{1}0.\text{1}1\text{,}p<.\text{0}0\text{1}$; RMSEA = .04; CFI = .93; TLI = .93; SRMR = .06] or listwise deletion [$\chi^{2}(\text{9}4\text{8})=\text{2}0\text{6}9.\text{4}6\text{,}p<.\text{0}0\text{1}$; RMSEA = .04; CFI = .94; TLI = .93; SRMR = .06]. The DIFFTEST revealed Model SST3 fitted worse than Model SST4, $\chi^{2}(10)=385.44,p<\mathrm{.001}$ for pairwise deletion and $\chi^{2}(10)=362.67,p<\mathrm{.001}$ for listwise deletion. In addition to the formal statistical test, RMSEA, CFI, TLI, and SRMR statistics also showed that Model SST4 was a better fitting model than Model SST3 for both pairwise and listwise deletion.

The standardized factor loadings on the general factor social skills from the three solutions of Model SST4 were very similar, with discrepancies all in the second-decimal place. However, discrepancies in the standardized factor loadings on domain-specific factors were larger across the three solutions, for Item 8 on the empathy subscale and for most items on the self-control subscale. Discrepancies in the correlations among domain-specific factors were also larger across the three solutions, with the greatest discrepancy being .18. Such discrepancies are likely due to the influence of missing data.

For interpretation, we focus on the parameter estimates in the MLR solution, which corrects for the nesting structure and accounts for missing data. Based on the MLR solution of Model SST4 (Table 3), the ECV was 0.74, meaning that the general factor social skills explained 74% of the common variance extracted from the teacher-report social skills items, with 26% of the common variance spread across the retained domain-specific factors. This finding supports a strong general factor. In terms of correlations among domain-specific factors (representing partial correlations corrected for common influences of the general factor), most of them corresponded to covariances that were not significantly different from zero. Specifically, the correlations were .92 between responsibility and cooperation,.36 between self-control and cooperation, and .60 between self-control and responsibility.

#### Teacher report: Problem behaviors.

S10–S11 Figs depict the CFA models fitted for the teacher-reported problem behaviors. We first fitted a single-factor CFA to all the teacher-report items on the problem behaviors scale excluding the reversely coded social skills items on the autism subscale (Model PBT1; a total of 30 items). The model fit based on ULSMV estimation was acceptable with either the default pairwise deletion [$\chi^{2}(\text{4}0\text{5})=1242.46\text{,}p<.\text{0}0\text{1}$; RMSEA = .05; CFI = .93; TLI = .92; SRMR = .07] or listwise deletion [$\chi^{2}(405)=1205.87\text{,}p<.\text{0}0\text{1}$; RMSEA = .05; CFI = .93; TLI = .93; SRMR = .07]. However, examination of the residuals for correlations revealed substantial underestimation of the correlations among items. Most of the residuals for correlations were among five out of seven items on the internalizing subscale: Items 56, 62, 64, 70, and 74. Such a trend was not observed for other problem behaviors subscales. There was also one negative residual for correlation less than −.20, between Items 51 (on the externalizing and the hyperactivity/inattention subscales) and 64 (on the internalizing subscale), indicating overestimation.

Next, we fitted an incomplete bi-factor model with a general factor problem behaviors on with all items loaded and a domain-specific factor, internalizing, for items on the internalizing subscale that was uncorrelated with the general factor (Model PBT2). The model fit of Model PBT2 based on ULSMV estimation was improved compared to Model PBT1, with either the default pairwise deletion [$\chi^{2}(\text{3}9\text{8})=859.13\text{,}p<.\text{0}0\text{1}$; RMSEA = .04; CFI = .96; TLI = .96; SRMR = .06] or listwise deletion [$\chi^{2}(398)=856.15\text{,}p<.\text{0}0\text{1}$; RMSEA = .04; CFI = .96; TLI = .96; SRMR = .06]. The DIFFTEST revealed that Model PBT1 fitted significantly worse than Model PBT2, $\chi^{2}(7)=157.79,p<\mathrm{.001}$ for pairwise deletion and $\chi^{2}(7)=146.61,p<\mathrm{.001}$ for listwise deletion.

We also explored a more complete bi-factor model with one more domain-specific factor for the hyperactivity/inattention subscale, identifying each factor using the item with the highest loading as the marker variable. All three solutions showed signs of overfactorization. Using ULSMV estimation with pairwise/listwise deletion, all freely estimated domain-specific factor loadings on the additional domain-specific factor, hyperactivity/inattention, were nonsignificant (Item 71 was used as the marker variable for this domain-specific factor) and the factor variance was nonsignificant, indicating overfactorization. Using MLR estimation, only two freely estimated domain-specific factor loadings (Items 53 and 65) on the additional domain-specific factor hyperactivity/inattention were significant; the factor variance was nonsignificant indicating overfactorization. Thus, we decided not to add the domain-specific factor for the hyperactivity/inattention subscale and to treat Model PBT2 as the final model.

Table 4 contains the model solutions for Model PBT2 using ULSMV estimation with pairwise/listwise deletion and MLR estimation, respectively. As illustrated, the standardized factor loadings from the three solutions of Model PBT2 were very similar, with discrepancies all in the second or third decimal place. For interpretation, we focus on the parameter estimates in the MLR solution, which corrects for the nesting structure and accounts for missing data. With the exception of two items in the internalizing problem subscale (Items 62 and 64, whose standardized loadings were .26 and .46, respectively), all items were strong indicators of the general factor problem behaviors, with standardized factor loadings ranging from .51 to .89. Most items on the internalizing problem subscale were also strong indicators of the domain-specific factor (standardized factor loadings were .30 and .16 for Items 68 and 76, respectively, but ranged from .52 to .73 for others). Of the 30 items, 28 (93.3%) had item *R*^2^ at or above .50, with the lowest item *R*^2^ of .35 for Item 48. The calculated ECV was .90 indicating a high proportion of variance explained by the general factor.

### Summary

Given that the total social skills score and total problem behaviors score are commonly used in the literature, we fitted single-factor and bi-factor models to the parents’ and teachers’ report of social skills and problem behaviors separately. The CFA results suggested adequate factorial validity of the overall factor. However, for the parent and teacher measure of social skills and the teacher measure of problem behaviors, some domain-specific factors explained the common variance among the items better. Important, not all SSIS-RS subscales were added as domain-specific factors to the bi-factor model (modified bi-factor model) and the additional domain-specific factors were different for the parent and teacher reports. Based on these findings, we can conclude the following: (a) consistent with the aim of the SSIS-RS scale, a general social skills or problem behaviors factor was found; (b) in all scales except for parent report of problem behaviors, certain domain-specific factors explained common variance among the items over and beyond the overall factor; and (c) the domain-specific factors to consider were different for the teacher and the parent reports.

For the parent-report problem behaviors scale, a single-factor model fitted well to the data, suggesting that the 33 items fit together well in measuring an overall problem behaviors construct. This indicates that the total score (sum or average of the 33 items) can be used to represent parents’ perception of their child’s problem behaviors.

For the other scales, bi-factor models fit better than single-factor models. This indicates that while the overall social skills or the problem behavior constructs explained much of the common variance among the items, additional variance may be explained by the subscales (specific factors). Specifically, for the parent-report social skills scale, although a good portion of variance (78%) was attributable to the overall social skills factor, the remaining variance could be uniquely (here “unique” means that it is unique from the overall factor) explained by cooperation, assertion, empathy, engagement, and self-control. Communication and responsibility were dropped from the list of subscales, meaning that while items in these subscales contributed to measuring the overall social skills, there was not enough uniqueness left to include the communication and responsibility factors as domain-specific factors in the bi-factor model.

In contrast, for the teacher-report social skills scale, responsibility was added, but at the same time communication and engagement factors were excluded from the bi-factor model. While 74% of the common variance among the items was attributed to the overall social skills factor, the remaining variance could be accounted for by the uniqueness of cooperation, assertion, responsibility, empathy, and self-control factors.

Finally, for the teacher-report problem behaviors scale, the internalizing factor contributed to explaining about 10% of the variance in the items while 90% of the variance was captured primarily by the overall problem behaviors factor.

## Discussion

The current study examined the factor structure of SSIS-RS scores with a Mexican American population using the Spanish parent and the English teacher versions of the instrument. Specifically, the study examined single- and bi-factor CFA models for the social skills scale and the problem behaviors scale separately for the Spanish parent version and the English teacher (preschool) version, respectively. Important, we were interested in the similarities/discrepancies in the factor structure between the parent and teacher ratings. For the Spanish parent version, the social skills underwent several models to support an incomplete bi-factor model with the removal of the communication and responsibility domain-specific factors. However, the problem behaviors scale was supported with a single-factor model. For the English teacher version, an incomplete bi-factor model was supported for both the social skills scale (removal of communication and engagement) and problem behaviors scale (removal of all, except the internalizing scale). These incomplete bi-factor models indicate that the removed factors did not provide additional information beyond the overall factor such as social skills or problem behaviors. Furthermore, the estimated ECVs suggest that a high proportion of the variance in the items can be explained by the general factor (78% and 74% for the parent and teacher measure of social skills, respectively, and 90% for the teacher measure of problem behaviors).

Clinicians typically use the total scores for each domain; however, these findings indicate that they would only need to examine the nonremoved subscales (e.g., internalizing disorders on teacher SSIS-RS problem behaviors) to gain additional information about a student’s needs for intervention. Important, instead of relying on composite scores (sum or average of items), our recommendation is to utilize structural equation modeling to identify and further study the relationship between these latent constructs and other variables. Composite scores not only contain measurement error, but they may also be a mixture of different constructs, including the overall social skills/problem behaviors and the domain-specific factors.

Since the initial investigation of the SSIS-RS psychometrics did not explore its factorial structure, it is possible that the factor structures identified in the current study are the best models for the measure when given to the Mexican American population [14]. The final models may vary based on the cultural or contextual interpretations of the parent or teacher expectations of low-income Mexican-ethnicity youth [42–43]. The different factor structures between parents and teachers might have originated from the different environments in which the parents and teachers observe the child. Parents observe their child at home and teachers observe the students at school. Some of the behaviors at home might be interpreted differently than when observed in a classroom. Another factor that might have contributed to the differences is the language of the SSIS-RS the parents (Spanish) and teachers (English) received. That is, the presented language might have triggered specific cultural beliefs and led to heterogenous factor structures explaining the observed responses. Further, Latinx children are raised with different values that may impact how their behaviors are exhibited to their parents and teachers or how they are perceived by parents and teachers [32]. For example, using the English teacher version of the problem behaviors scale, the clinician would look at the overall problem scale score to understand the child’s behavior and then investigate the internalizing scale to link an intervention if the subscale was elevated. If the overall problem scale was elevated and the internalizing subscale was not, the clinician would target overall problem behaviors within the intervention. Culturally, clinicians may interpret the internalizing behaviors as a unique factor compared to the overall problem behaviors demonstrated by Mexican American students [44,45]. In contrast, parent reports might not distinguish unique internalizing symptoms of their child and, therefore, only the overall problem behavior score would be interpretable. In sum, our findings highlight the importance of using caution when interpreting the SSIS-RS with ethnic populations that differ from the norm sample.

Our findings also revealed some similarities in factor structure between the different informants. For both parents and teachers, a bi-factor model fitted adequately to the social skills domain data. However, there were discrepancies in the specific domain-specific factors between the parents and teachers. These discrepancies do not necessarily diminish the validity of the SSIS-RS; rather, they emphasize the importance of cross-informant perspectives. Utilizing multiple informants, the SSIS-RS can be used to learn different aspects of the children’s behaviors across settings where expectations may vary.

### Limitations and directions for future research

The population in this study was unique, and the results, therefore, may not be generalizable to other diverse populations. The parent informants were Latinx mothers who were low SES with limited educational backgrounds of primarily Mexican origin and who spoke Spanish in the home. Teacher informants, also primarily Mexican in origin, were of higher income and more education. Moreover, although there were multiple parent and teacher informants—thus reducing the potential for informant bias—it is altogether possible that both teachers and parents, each lacking complete knowledge of the child’s behavior across all settings (i.e., limited proximity to information), may have been biased in their reporting of the children’s behavior. As a result, the presence of informant bias may limit the generalizability of the findings, since the reported behaviors might not fully represent the child’s conduct across diverse environments or in the broader population.

Additionally, our sample size was relatively small compared to the norming sample used in the initial study. Therefore, future studies should use a larger, diverse sample of Latinx students beyond the low-SES Mexican American students in the current study. Further, given that most of the teachers in our study were Latinx, future studies should consider the effects of the diversity of raters on findings.

Another limitation of the study is that the tested CFA models were heavily influenced by the original conceptualization of the domains and constructs [14]. Other factor models might fit the data as well as or better. For example, Gresham et al. [33] utilized the SSIS to measure Collaborative on Academic Social Emotional Learning (CASEL) skills. Their proposed factor structure model based on the SEL factors fitted adequately to the parent and student report data but displayed a mediocre fit to the teacher report data.

Finally, the present study only concentrated on the factorial validation of the SSIS-RS scores. Additional psychometric properties such as convergent/divergent validity or criterion validity should be investigated for the Mexican American population. Especially, future studies should investigate the relationship between the domain-specific factors and theoretically related (or unrelated) constructs of interest. Another recommendation for future study is to utilize qualitative data to understand parents’ and teachers’ thoughts about the children’s behavior and their understanding of the SSIS-RS questions. Such study could clarify why discrepancies were found between the factor structures of the SSIS-RS scores of parents vs. teachers.

The current study is the first to examine the factor structure of the SSIS-RS in a Mexican American population. While adequate psychometric properties were identified, they varied slightly from the proposed scale models of the SSIS-RS [14]. As mentioned, this variation may be due to the cultural differences of our study’s population compared to the initial sample [14]. The models used in our study provide insight for clinicians and researchers using scale scores and subscale scores of the SSIS-RS. Specifically, for the parent version in Spanish, the communication and responsibility subscales may not contribute clinically relevant information beyond the information provided by the social skills scale score. Similarly, for the teacher version, the communication and engagement subscales did not provide additional information on top of the social skills scale score. Further, most of the subscales for the teacher version problem behaviors scale did not provide important information about children’s problem behaviors whereas the internalizing behaviors subscale provided unique information. Thus, the internalizing behavior subscale score could provide additional information to a clinician for tailoring the target of intervention for children with elevated internalizing behavior scores.

Clinicians can use the findings from the current study to provide more individualized interpretations and recommendations within their evaluation reports. Specifically, they can use this guidance to target the unremoved subscales for intervention to improve the youth’s social skills and reduce their problem behaviors. These findings suggest that culture influences how youth’s behaviors may be exhibited or interpreted by their parents and teachers.

In recent decades, there has been a marked shift in testing practices among school psychologists, most notably in the form of a sharp increase in the use of behavior rating scales [15], which offer a cost-effective and efficient method for measuring a broad range of skills, including social skills. Given the key role of social skills in child development, their accurate assessment is paramount as deficiencies in social skills can serve as indicators of poor overall functioning, underscoring the importance of comprehensively evaluating social abilities for a better understanding of positive child development.

In short, accurate assessment of social skills is crucial for understanding positive child development. While the SSIS-RS continues to be a popular multi-rater assessment for social skills, few studies have examined the psychometric properties of the most recent iteration of the SSIS-RS with diverse populations as in our study. Moreover, since its development did not include an exploratory factorial investigation, the current study findings may also apply to other diverse populations as well as the overall general population.

## Supporting information

S1 Fig Parent report social skills model SSP1: Single-factor model. (DOCX)

S2 Fig Parent report social skills model SSP2: Original bi-factor model. (DOCX)

S3 Fig Parent report social skills model SSP3: Modified bi-factor model. (DOCX)

S4 Fig Parent report social skills model SSP4: Final selected modified bi-factor model. (DOCX)

S5 Fig Parent report problem behaviors model PBP1: Final selected single-factor model. (DOCX)

S6 Fig Teacher report social skills model SST1: Single-factor model. (DOCX)

S7 Fig Teacher report social skills model SST2: Original bi-factor model. (DOCX)

S8 Fig Teacher report social skills model SST3: Modified bi-factor model. (DOCX)

S9 Fig Teacher report social skills model SST4: Final selected modified bi-factor model. (DOCX)

S10 Fig Teacher report problem behaviors model PBT1: Single-factor model. (DOCX)

S11 Fig Teacher report problem behaviors model PBT2: Final selected modified bi-factormodel. (DOCX)
