# Supplementary material for: An examination of the Social Skills Improvement System-Rating Scale (SSIS-RS) teacher and parent forms factor structure in a sample of Mexican American preschool-aged children
Source: PLoS One. 2025 Aug 20;20(8):e0329576. doi: 10.1371/journal.pone.0329576 (PMC12367192; doi:10.1371/journal.pone.0329576)
Supplement: S2 Fig — (DOCX) [file pone.0329576.s002.docx]

**Figure 2**

*Parent Report Social Skills Model SSP2: Original Bi-Factor Model*

*Note.* The dashed part of the model used heuristics to indicate multiple elements specified in a similar way. Note that residuals are omitted from the model for simplicity. There were 7 domain-specific factors specified.

Item 1 for F_1_

Item *k* for F_1_

Item *K* for F_1_

Item 1 for F*_J_*

Item *p* for F*_J_*

Item *P* for F*_J_*

Item *l* for F*_j_*
