# Supplementary material for: An examination of the Social Skills Improvement System-Rating Scale (SSIS-RS) teacher and parent forms factor structure in a sample of Mexican American preschool-aged children
Source: PLoS One. 2025 Aug 20;20(8):e0329576. doi: 10.1371/journal.pone.0329576 (PMC12367192; doi:10.1371/journal.pone.0329576)
Supplement: S4 Fig — (DOCX) [file pone.0329576.s004.docx]

**Figure 4**

*Parent Report Social Skills Model SSP4: Final Selected Modified Bi-Factor Model*

Item 1

Item *k*

Item *K*

Item 37

Item *p*

Item *P*

Item *l* for F*_j_*

0

*Note.* The dashed part of the model used heuristics to indicate multiple elements specified in a similar way. Note that residuals are omitted from the model for simplicity. From Model SSP3 (Figure 3), the covariances between domain-specific factors were allowed to be freely estimated.
