# Supplementary material for: An examination of the Social Skills Improvement System-Rating Scale (SSIS-RS) teacher and parent forms factor structure in a sample of Mexican American preschool-aged children
Source: PLoS One. 2025 Aug 20;20(8):e0329576. doi: 10.1371/journal.pone.0329576 (PMC12367192; doi:10.1371/journal.pone.0329576)
Supplement: S8 Fig — (DOCX) [file pone.0329576.s008.docx]

**Figure 8**

*Teacher Report Social Skills Model SST3: Modified Bi-Factor Model*

Item 1

Item *k*

Item *K*

Item 42

Item *p*

Item *P*

Item *l* for F*_j_*

0

*Note*. The dashed part of the model used heuristics to indicate multiple elements specified in a similar way. Note that residuals are omitted from the model for simplicity. From Model SST2 (Figure 7), two domain-specific factors (communication & engagement) were dropped. A total of five remaining domain-specific factors were specified (i.e., cooperation, responsibility, assertion, empathy, & self-control). Additionally, an ill-behaving factor loading for Item 42 (item for responsibility factor) was constraint to zero.
