# Supplementary material for: An examination of the Social Skills Improvement System-Rating Scale (SSIS-RS) teacher and parent forms factor structure in a sample of Mexican American preschool-aged children
Source: PLoS One. 2025 Aug 20;20(8):e0329576. doi: 10.1371/journal.pone.0329576 (PMC12367192; doi:10.1371/journal.pone.0329576)
Supplement: S10 Fig — (DOCX) [file pone.0329576.s010.docx]

**Figure 10**

*Teacher Report Problem Behaviors Model PBT1: Single-Factor Model*

Item 1

Item 2

Item 3

Item 28

Item 29

Item 30

Item *p*

*Note*. The dashed part of the model used heuristics to indicate multiple items specified in a similar way. Note that residuals are omitted from the model for simplicity.
