# Supplementary material for: An examination of the Social Skills Improvement System-Rating Scale (SSIS-RS) teacher and parent forms factor structure in a sample of Mexican American preschool-aged children
Source: PLoS One. 2025 Aug 20;20(8):e0329576. doi: 10.1371/journal.pone.0329576 (PMC12367192; doi:10.1371/journal.pone.0329576)
Supplement: S11 Fig — (DOCX) [file pone.0329576.s011.docx]

**Figure 11**

*Teacher Report Problem Behaviors Model PBT2: Final Selected Modified Bi-Factor Model*

Item 1

Item 2

Item 3

Item 1 for Int.

Item *m* for int.

Item *M* for int.

Item *p*

*Note*. The dashed part of the model used heuristics to indicate multiple elements specified in a similar way. Note that residuals are omitted from the model for simplicity. Only one domain-specific factor, internalizing was specified above and beyond the general problem behaviors factor.
